# Correlated species differences in the effects of cannabinoid ligands on anxiety and on GABAergic and glutamatergic synaptic transmission

**DOI:** 10.1111/j.1460-9568.2007.05476.x

**Published:** 2007-04-01

**Authors:** J Haller, F Mátyás, K Soproni, B Varga, B Barsy, B Németh, É Mikics, T F Freund, N Hájos

**Affiliations:** 1 Institute of Experimental Medicine, Department of Behavioral Neurobiology 1450 Budapest, PO Box 67, Hungary; 2 Institute of Experimental Medicine, Department of Cellular and Network Neurobiology Budapest, Hungary; 3 Richter Gedeon Pharmaceuticals Budapest, Hungary

**Keywords:** AM-251, EPSC, IPSC, mice, rats, WIN-55, 212

## Abstract

Cannabinoid ligands show therapeutic potential in a variety of disorders including anxiety. However, the anxiety-related effects of cannabinoids remain controversial as agonists show opposite effects in mice and rats. Here we compared the effects of the cannabinoid agonist WIN-55,212 and the CB1 antagonist AM-251 in CD1 mice and Wistar rats. Special attention was paid to antagonist–agonist interactions, which had not yet been studied in rats. In mice, WIN-55,212 decreased whereas AM-251 increased anxiety. The antagonist abolished the effects of the agonist. In contrast, WIN-55,212 increased anxiety in rats. Surprisingly, the antagonist potentiated this effect. Cannabinoids affect both GABAergic and glutamatergic functions, which play opposite roles in anxiety. We hypothesized that discrepant findings resulted from species differences in the relative responsiveness of the two transmitter systems to cannabinoids. We investigated this hypothesis by studying the effects of WIN-55,212 on evoked hippocampal inhibitory and excitatory postsynaptic currents (IPSCs and EPSCs). IPSCs were one order of magnitude more sensitive to WIN-55,212 in mice than in rats. In mice, IPSCs were more sensitive than EPSCs to WIN-55,212. This is the first study showing that the relative cannabinoid sensitivity of GABA and glutamate neurotransmission is species-dependent. Based on behavioural and electrophysiological findings, we hypothesize that WIN-55,212 reduced anxiety in mice by affecting GABA neurotransmission whereas it increased anxiety in rats via glutamatergic mechanisms. In rats, AM-251 potentiated this anxiogenic effect by inhibiting the anxiolytic GABAergic mechanism. We suggest that the anxiety-related effects of cannabinoids depend on the relative cannabinoid responsiveness of GABAergic and glutamatergic neurotransmission.

## Introduction

The identification of cannabinoid receptors and their endogenous ligands triggered an exponential growth in studies exploring the endocannabinoid system and its regulatory functions in health and disease (Pacher *et al*., 2006). Cannabinoid ligands have therapeutic potential in a wide range of pathological conditions, including anxiety. However, the anxiety-related effects of cannabinoids are controversial, especially when data obtained in different species are compared.

In mice, anxiety is increased by both the genetic disruption of the CB1 receptor and its pharmacological blockade by AM-251 (Haller *et al*., 2002, 2004a, 2004b; Maccarrone *et al*., 2002; Martin *et al*., 2002; Uriguen *et al*., 2004; Rodgers *et al*., 2005; Patel & Hillard, 2006). In line with the effects of CB1 blockade, cannabinoid agonists (Δ9-tetrahydrocannabinol, WIN-55,212, and CP 55 940) decreased anxiety in mice (Berrendero & Maldonado, 2002; Valjent *et al*., 2002; Haller *et al*., 2004b; Patel & Hillard, 2006). Although conflicting data are reported with the antagonist SR-141716A (Haller *et al*., 2002; Rodgers *et al*., 2003; Griebel *et al*., 2005; Patel & Hillard, 2006), taken together, data suggest that cannabinoids mediate anxiolysis in mice. Antagonists and agonists were rarely applied together, but two studies suggest that the former abolish the effects of the latter, i.e. the effects are mediated by the CB1 receptor (Berrendero & Maldonado, 2002; Haller *et al*., 2004b).

In contrast to mice, nonsedative doses of CB1 agonists (AM-411, CP-55,940 and HU-210) increased anxiety in the elevated plus-maze, social interaction, open field and defensive withdrawal tests in Wistar, Sprague–Dawley, hooded Lister and Long–Evans rats (Rodriguez de Fonseca *et al*., 1996; Giuliani *et al*., 2000; Arevalo *et al*., 2001; Marin *et al*., 2003; Genn *et al*., 2004; Hill & Gorzalka, 2004; Marco *et al*., 2004; McLaughlin *et al*., 2005). Two studies suggest that the antagonist SR-141716A is also anxiogenic in rats (Arevalo *et al*., 2001; Navarro *et al*., 1997). Surprisingly, antagonist–agonist interactions were not studied in this species. Although the lack of such studies casts doubt on the conclusion, data obtained with agonists suggest that cannabinoids mediate anxiogenic effects in rats.

In the present study, we compared the effects of the cannabinoid agonist WIN-55,212 and the CB1 antagonist AM-251 in CD1 mice and Wistar rats, strains that have been frequently studied. Special attention was paid to antagonist–agonist interactions, which are largely unknown in rats. The impact of experimental conditions was also studied. Our findings confirmed the species-dependent effects of cannabinoids; moreover, we revealed a paradoxical antagonist–agonist interaction in rats. As cannabinoids have been shown to affect both glutamatergic and gamma-aminobutyric acid (GABA) ergic mechanisms (Hájos *et al*., 2001; Hájos & Freund, 2002; Piomelli, 2003), which play opposite roles in anxiety (Millan, 2003), we hypothesized that the species-dependent effects of cannabinoids on anxiety depend on differences in the cannabinoid sensitivity of glutamatergic and GABAergic neurotransmission. This hypothesis was investigated by comparing the effects of WIN-55,212 on hippocampal excitatory and inhibitory postsynaptic currents in mice and rats.

## Materials and methods

### Experimental animals

Subjects were CD1 mice and Wistar rats obtained from Charles River Laboratories (Budapest, Hungary). Approximately two-month-old mice (body weight was ∼ 35 g) and two-month-old rats (body weight was ∼ 300 g) were used. Animals were acclimatized to local conditions for at least 1 week. Food and water were available *ad libitum*. Temperature and humidity were 23 ± 2 °C and 60 ± 10%, respectively. Experiments were conducted in both the light and dark phases of the day. Mice establish strong dominance hierarchies, and social status affects anxiety in this species (Ferrari *et al*., 1998). To avoid confounds from social status, subjects were kept in individual cages for 2 weeks prior to experimentation. The impact of housing conditions was also studied in rats.

Experiments were carried out in accordance with the European Communities Council Directive of 24 November 1986 (86/609/EEC) and were reviewed and approved by the Animal Welfare Committee of the Institute of Experimental Medicine.

### Behavioural studies

Both the mouse and the rat plus-maze were made of dark grey painted wood. The dimensions of the apparatus were adapted to the size of the species (mice: arm length 30 cm, arm width 7 cm, wall height 30 cm and platform height 80 cm; rats: arm length 50 cm, arm width 17 cm, wall height 30 cm and platform height 80 cm). Open arms were surrounded by 0.3- and 0.5-cm-high ledges in mice and rats, respectively. Subjects were placed in the central area of the apparatus with head facing a closed arm. Exposure lasted 5 min. Closed-arm entries were considered indicators of locomotor activity whereas open-arm exploration was used as a measure of anxiety. Open arm exploration was characterized by two variables: percentage time spent in the open arm, and percentage open arm entries (100 × open arm entries/total arm entries).

It has been shown that mice are sensitive to cannabinoid effects only when the plus-maze is brightly lit (Haller *et al*., 2004a). Therefore, mice were tested under intense light (four white lamps of 40 W each; ∼ 200 lux). Similar illumination is strongly anxiogenic in rats. Therefore, the rat plus-maze was illuminated by a red lamp of 40 W (∼ 1 lux) in the first experiments. Separate studies were run to investigate the impact of illumination. The effects of the diurnal cycle were also investigated.

WIN-55,212 and AM-251 were obtained from Tocris Cookson Ltd (UK), and were dissolved in dimethyl sulphoxide (DMSO) first, and diluted with 0.4% methylcellulose (in saline) to the final volume. The concentration of DMSO was < 1.5% in the final solution. Controls received DMSO-containing methylcellulose in similar concentrations. Combined treatments involved the i.p. injection of a mixture of the two compounds (i.e. one injection was given). Chlordiazepoxide (Sigma, Budapest, Hungary) was dissolved in 0.4% methylcellulose, and injected i.p.

Experiments 1 and 2 investigated the effects of the CB1 antagonist AM-251 in mice (*N* = 11 or 12 per group) and rats (*N* = 9 or 10 per group). These experiments were conducted according to the protocols used earlier in our laboratory (mice: light phase of the day, social isolation, strongly illuminated plus-maze; rats: dark phase of the day, group housing, dimly illuminated plus-maze). The impact of experimental conditions was studied in experiments 5–7.

Experiment 3 evaluated in mice the effects of the cannabinoid agonist WIN-55,212 alone or in combination with the antagonist AM-251. Two experiments were performed. Experiment 3a assessed the effect of WIN-55,212 (1 and 3 mg/kg; N = 10 per group). In Experiment 3b, the effects of the large WIN-55,212 dose (3 mg/kg) was investigated alone or in combination with a low dose of the antagonist that was without effect on its own (0.5 mg/kg; *N* = 10 per group).

The effects of WIN-55,212 in rats were studied in Experiment 4. Experiment 4a (*N* = 8 per group) assessed the effects of a small and a large dose (0.1 and 1 mg/kg, respectively). As the effects were opposite to those seen in mice, intermediate doses (0.3 and 0.5 mg/kg; *N* = 12) and a larger dose (3 mg/kg; *N* = 8) were also investigated (Experiment 4b). Thus, the effects of the agonist were studied over a large dose range (0.1–3 mg/kg).

Experiments 5–7 were performed to evaluate the impact of experimental conditions on cannabinoid responsiveness in rats. In Experiment 5, we administered WIN-55,212 in the light phase of the day to rats maintained in social isolation (*N* = 10 per group). Plus-maze testing was performed under dim red light. Experiment 6 assessed the effects of the same agonist during the light phase in rats maintained in social isolation, the plus-maze testing being performed under high white light (*N* = 9 or 10 per group). In Experiment 7, we studied the effects of AM-251 during the light phase of the day in rats maintained in social isolation (*N* = 9 per group).

The antagonist–agonist interaction in rats was examined in Experiment 8. We treated rats with 0.3 and 1 mg/kg WIN-55,212 alone or in combination with 1 mg AM-251 (*N* = 8 per group). It is noteworthy that AM-251 alone did not affect behaviour in rats.

Experiment 9. To rule out the possibility that differences seen with cannabinoid ligands were due to intrinsic species differences in response to anxiolytics, we studied the response of both mice and rats to the anxiolytic chlordiazepoxide in the elevated plus-maze. Conditions were similar with those employed in Experiments 1 and 2.

### Electrophysiology

Horizontal slices of the hippocampus (400 µm thick) were prepared from Wistar rats (14–17 days old) or 350-µm-thick sections from CD1 mice (15–25 days old) as described elsewhere (Hájos & Freund, 2002). Animals were anesthetized by isofluran inhalation. Slices were incubated for ≥ 1 h in ACSF at room temperature in an interface-type chamber, and then transferred to a submerged-type recording chamber. Whole-cell patch-clamp recordings were obtained at 30–32 °C from CA1 pyramidal cells visualized by infrared DIC videomicroscopy (Zeiss Axioscope, Germany) using a flow rate of 3–4 mL/min in a slice chamber optimized for laminar flow to ensure the stability of the amplitude of evoked currents. The extracellular solution (artificial cerebrospinal fluid; ACSF) had a composition of (in mm) NaCl, 126; KCl, 2.5; NaHCO_3_, 26; CaCl_2_, 2; MgCl_2_, 2; NaH_2_PO_4_, 1.25; and glucose, 10; the intrapipette solution contained (in mm) CsCl, 80; Cs-gluconate, 60; NaCl, 3; MgCl_2_, 1; HEPES, 10; Mg-ATP, 2; and QX-314, 5 (pH 7.2–7.3 adjusted with CsOH; osmolarity 275–290 mOsm). To isolate the excitatory (E) postsynaptic currents (PSCs), slices were perfused with ACSF containing 70–100 µm picrotoxin to block GABA_A_ receptor-mediated transmission. To record inhibitory postsynaptic currents (IPSCs), the ACSF contained 2–3 mm kynurenic acid to eliminate ionotropic glutamatergic transmission. Recordings were made at a holding potential of −65 mV. Electrical stimulation was delivered, via a theta glass pipette (Sutter Instruments) containing ACSF, at 0.1 Hz using a Supertech timer and isolator (Supertech LTD, Pécs, Hungary). To evoke EPSCs, the pipette was placed into the stratum radiatum while IPSCs were evoked by an electrode placed into the stratum pyramidale. Access resistances (between 4 and 18 MΩ, compensated 65–70%) were frequently monitored and remained constant (±20%) during the period of analysis. Signals were recorded with a Multiclamp 700A (Axon Instruments, CA, USA), filtered at 2 kHz, digitized at 6–10 kHz (PCI-6024E A/D board; National Instruments, Austin, TX, USA) and analysed off-line with the EVAN program (courtesy of I. Mody, UCLA, CA, USA). The rising and the decaying phases of averaged PSCs were fitted with a single exponential function.

Only the effects of WIN-55,212 were studied, as AM-251 *per se* does not affect postsynaptic currents (Kreitzer & Regehr, 2001; Nakatsuka *et al*., 2003; Hájos *et al*., 2004). We have shown earlier that HCl solvent ensures a greater stability of the response in electrophysiological experiments (Hájos & Freund, 2002). Therefore, WIN-55,212 (Sigma) was dissolved in 0.1 n HCl to give a 20 mm stock solution. From this stock solution, the final dilution of WIN-55,212 was made in ACSF containing either kynurenic acid or picrotoxin under constant stirring, and the prepared solution was bath-applied. The drug was superfused until the maximal effect was seen. Usually this took 6–8 min. To quantify the drug effects, control PSC amplitudes in a 2- to 3-min time window were compared with those measured after 10 min drug application for the same period of time. To avoid the possible effect of the change in pH, we added the same amount of HCl to the control solution. Only those experiments were included that had stable amplitude for at least 10 min before drug application. After each experiment, the tubing made of Teflon was washed with ethanol for 10 min and with ACSF for 15 min. Each data point represents the mean ± SEM of the maximal inhibition of the evoked PSCs (*n* = 3–5).

One animal per day was studied, and the species was randomly chosen for the particular day. Similarly in both species, three animals each were tested with the low and high concentrations, and four or five animals with intermediate concentrations. On average, three recordings per animal were performed and two different concentrations were examined.

### Immunocytochemistry

Male Wistar rats and CD1 mice were deeply anaesthetized with Equithesin (0.3 mL/100 g), and perfused first with physiological saline (2 min), and then with 100 or 400 mL (mice and rats, respectively) fixative containing 0.05% glutaraldehyde (TAAB Laboratory Equipment, Berks, UK), 4% paraformaldehyde (Sigma) and 0.2% picric acid in 0.1 m phosphate buffer (PB; pH 7.4) for 30 min. After fixation, brains were removed from the skull and were postfixed in the same fixative for 1 h. Blocks containing the dorsal hippocampus were sectioned on a Vibratome at 50 µm, then washed extensively in PB. Sections from the left and right hemispheres were incubated separately. All sections were incubated first in a blocking solution containing 3% bovine serum albumin (Sigma) and 0.5% Triton X-100 (Sigma) for 1 h. The CB1 receptors were stained with the antibody (diluted 1 : 1000) for 2 days at 4 °C; it was raised against a glutathione S-transferase fusion protein containing the entire C-terminus of the rat CB1 receptor. It has been shown that this antibody reliably labels receptors located on GABAergic cells but does not label CB1 receptors located on glutamatergic neurons (Katona *et al*., 1999, 2001; Hájos *et al*., 2000; Mátyás *et al*., 2004; Bodor *et al*., 2005; Nyíri *et al*., 2005). The specificity of the antisera and the method was confirmed in CB1-knockout (-KO) animals, as in an earlier study of our lab (Hájos *et al*., 2000). All the washing steps (3 × 10 min) and the dilution of the antisera were performed in 50 mm Tris-buffered saline (TBS; pH 7.4). Sections were then incubated in Alexa 594-conjugated goat antirabbit IgG (1 : 200; Molecular Probes, Eugene, OR, USA) for 2 h at room temperature. Sections were again washed in TBS, mounted in Vectashield to retard fluorescence fading, and then cover-slipped.

Five sections each from three mice and three rats were paired in such a way that sections from the two species were processed together in the same vials. The immunostaining protocol was thus carried out in 15 vials in parallel, and analysed at the same time to avoid bleaching of the fluorescent dye.

One image from randomly selected areas of both surfaces (areas spanning from the CA1 alveus to the middle of the dentate hilus, at a width of ∼ 200–300 µm) of five sections each (mice and rats) from the hippocampi were captured at a depth of 3 µm from the section surface on an Olympus FV300-BX confocal laser-scanning microscope with a 40× objective with a numerical aperture of 3. We used the publicly available morphometry program ImageJ to measure the density of CB1 labelling in the distinct layers of CA1 and dentate gyrus. To make our data simpler to understand, we inverted the grayness values. Thus, the value 0 indicated no staining (white) while the value 255 referred to the darkest staining (black), i.e. higher grayness corresponds to stronger staining. We analysed the mean grey value of each image. Data from left and right sections, as well as upper and lower surface images of the same species, did not differ significantly and were pooled. Thus, a total of 120 images from the dentate gyrus and CA1 of mice and rats were analysed and resulted in 30 pooled images (15 each of mice and rats), which were then statistically compared.

### Statistics

Behavioural, electrophysiological and immunocytochemical data were analysed using anova. The homogeneity of variances was tested by the Levene test, and data underwent square-root transformation where necessary. The Fisher LSD test was used for pairwise comparisons.

## Results

### The effects of the CB1 antagonist AM-251 in mice and rats

**Fig. 1:**
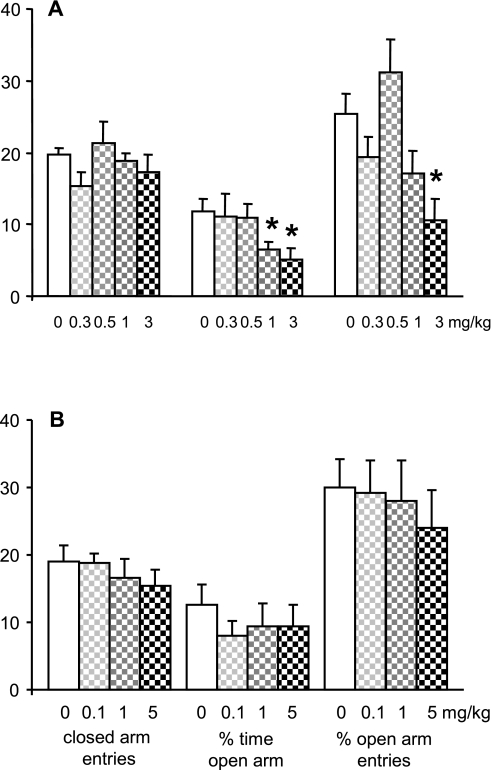
AM-251 increased anxiety-like behaviour in (A) mice without affecting it in (B) rats. *P < 0.05, at least, compared with vehicle control. Additional experiments showed that the slight (not significant) effects seen in rats were due to chance (see Fig. 4).

In mice, the CB1 receptor antagonist AM-251 had a clear anxiogenic effect without affecting locomotion (Fig. 1A). Closed-arm entries were not affected significantly (*F*_4,65_ = 1.41; *P* > 0.2), whereas the time spent in the open arms and percentage of open-arm entries were marginally and significantly reduced, respectively (*F*_4,65_ = 2.42, *P* = 0.057; *F*_4,65_ = 4.79, *P* < 0.003). Open-arm entries were also significantly reduced, from 7.30 ± 1.09 seen in controls to 2.42 ± 0.7 in the group treated with 3 mg/kg AM-251 (*F*_4,65_ = 5.41; *P* < 0.001).

In contrast, rats responded poorly to AM-251; a wider dose range (0.1–5 mg/kg vs. 0.3–3 mg/kg) failed to induce significant changes in plus-maze behaviour (closed-arm entries, *F*_3,34_ = 0.76, *P* < 0.5; percentage of open-arm time, *F*_3,34_ = 0.46; *P* < 0.7; percentage of open-arm entries, *F*_3,34_ = 0.48, *P* < 0.7; Fig. 1B).

### The effects of the CB1 agonist WIN-55,212 in mice

**Fig. 2:**
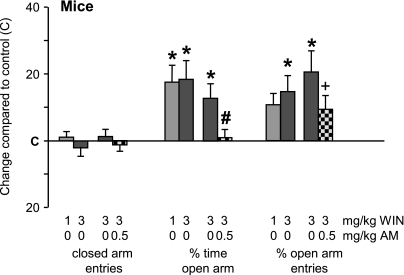
The cannabinoid agonist WIN-55,212 (WIN) decreased anxiety-like behaviour in the mouse elevated plus-maze and the effect was abolished by the specific CB1 receptor antagonist AM-251 (AM). Data are derived from two different experiments in which statistical analysis was run separately (see Results). For clarity, data are presented here as differences from vehicle control. *P < 0.05, at least, vs. vehicle control; ^#^*P* < 0.05 vs. WIN-55,212, 3 mg/kg; ^+^, marginally significant difference (0.05 < ^+^*P <* 0.1) from WIN-55.212, 3 mg/kg.

The cannabinoid agonist WIN-55,212 produced clear anxiolytic effects in mice (Fig. 2). Locomotor behaviour was not affected (*F*_2,27_ = 0.64, *P* < 0.5). In contrast, open-arm exploration was increased (*F*_2,27_ = 4.94, *P* < 0.02 and *F*_2,27_ = 2.94, *P* < 0.07, for duration and percentage of open-arm visits, respectively). In Experiment 3b, the agonist WIN-55,212 again reduced anxiety without affecting locomotion (closed-arm entries, *F*_2,27_ = 0.31; *P* > 0.7; percentage of open-arm time, *F*_2,27_ = 5.23; *P* < 0.01; percentage of open-arm entries, *F*_2,27_ = 4.51; *P* < 0.02). The low dose of AM-251 (0.5 mg/kg) abolished the effects of the agonist on percentage of open-arm time whereas the increase in percentage of open-arm entries was marginally reduced (*P <* 0.1). The group treated with WIN-55,212 and AM-251 did not differ significantly from controls in either variable.

### The effects of WIN-55,212 in rats

**Fig. 3:**
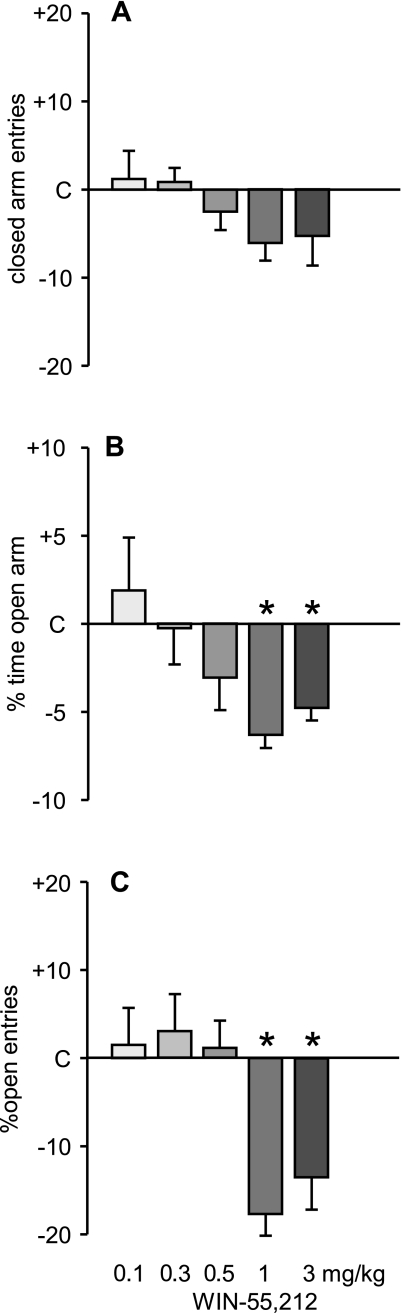
The cannabinoid agonist WIN-55,212 increased anxiety-like behaviour in the rat elevated plus-maze. (A) Effects on locomotion (closed-arm entries); (B and C) effects on anxiety (percentage open-arm time and percentage open-arm entries, respectively). Data are expressed as differences from control (C). *P < 0.05, at least, vs. control.

The effects of WIN-55,212 in rats were opposite to those seen in mice (Fig. 3). In Experiment 4a, the 0.1 mg/kg dose had no significant effect on behaviour whereas the larger dose (1 mg/kg) was clearly anxiogenic, with no significant effect on locomotion (closed-arm entries, *F*_2,21_ = 1.44, *P* < 0.3; percentage of open-arm time, *F*_2,21_ = 3.29, *P* < 0.05; percentage of open-arm entries, *F*_2,21_ = 6.34, *P* < 0.007). In Experiment 4b, intermediate doses (0.3 and 0.5 mg/kg) had minor effects on behaviour whereas a larger dose (3 mg/kg) was also anxiogenic (closed-arm entries, *F*_1,13_ = 1.20, *P* < 0.3; percentage of open-arm time, *F*_1,13_ = 5.56, *P* < 0.04; percentage of open-arm entries, *F*_1,13_ = 7.10, *P* < 0.02).

**Fig. 4:**
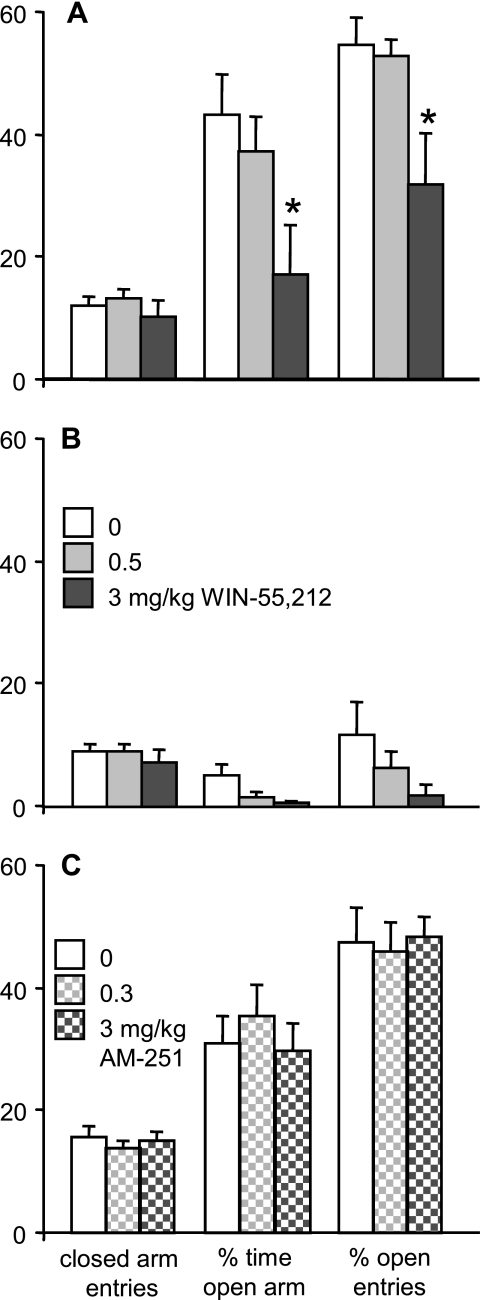
Testing conditions had no impact on the effects of cannabinoid ligands on anxiety in rats. WIN-55,212 remained anxiogenic in individually housed rats tested in the light phase of the day under both (A) dim and (B) intense light conditions. Additional data on the behaviour of rats in the latter test are included in Results. The CB1 antagonist AM-251 had no effects on anxiety in individually housed rats tested in the light phase of the day (C). *P < 0.05 vs. control.

Experimental conditions affected plus-maze behaviour (e.g. intense light increased anxiety) but did not influence the effects of cannabinoids. WIN-55,212 was clearly anxiogenic in individually housed rats that were assessed during the passive (light) phase of the day under dim light (closed-arm entries, *F*_2,27_ = 0.60, *P* < 0.6; percentage of open-arm time, *F*_2,27_ = 3.85, *P* < 0.0033; percentage of open-arm entries, *F*_2,27_ = 4.68, *P* < 0.017; Fig. 4A). Open-arm exploration was very low when socially isolated rats were studied during the light phase of the day and were tested under intense light (Fig. 4B). This precluded the expression of significant anxiogenic effects. The nonsignificant changes, however, were consistent with increased anxiety. This conclusion was supported by significant changes in the time spent in the closed arms and the central area. At 3 mg/kg, WIN-55,212 increased the percentage of closed-arm time (controls, 54.5 ± 6.1; 0.3 mg/kg WIN-55,212, 53.4 ± 5.1; 3 mg/kg WIN-55,212, 73.5 ± 6.0%; *F*_2,26_ = 3.74, *P* < 0.037) and decreased the percentage of time spent in the central area (a less safe compartment of the plus-maze; controls, 40.5 ± 4.6; 0.3 mg/kg WIN-55,212, 45.2 ± 4.8; 3 mg/kg WIN-55,212, 25.9 ± 5.7%; *F*_2,26_ = 3.86, *P* < 0.033). The preference for the safest compartment (closed-arm) and the avoidance of the less safe central area suggest that the compound was anxiogenic under these conditions as well.

AM-251 did not affect behaviour when tested in the light phase of the day (closed-arm entries, *F*_2,24_ = 0.38; *P* < 0.7; percentage of open-arm time, *F*_2,24_ = 0.42; *P* < 0.7; percentage of open-arm entries, *F*_2,24_ = 0.06; *P* < 09; Fig. 4C).

### AM-251–WIN-55,212 interactions in rats

**Fig. 5:**
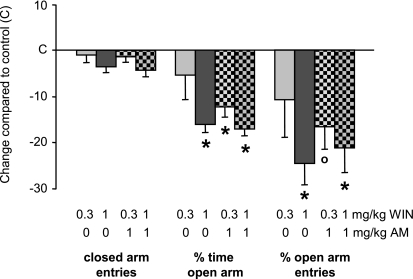
The CB1 antagonist AM-251 potentiated the effects of the cannabinoid agonist WIN-55,212 on anxiety-like behaviour in rats. Data are expressed as differences from control (C). The low dose of the agonist (ineffective *per se*) became anxiogenic when administered together with the antagonist. *P < 0.05, at least, vs. vehicle control.

In line with the findings presented above, 0.3 mg/kg WIN-55,212 had no effects on plus-maze behaviour, whereas 1 mg/kg was anxiogenic (Fig. 5; percentage of open-arm time, *F*_4,35_ = 4.90; *P* < 0.003; percentage of open-arm entries, *F*_4,35_ = 3.01; *P* < 0.03). Closed-arm entries were not affected. AM-251 did not prevent the effects of 1 mg/kg WIN-55,212. Surprisingly, the low dose of the agonist (ineffective *per se*) significantly increased anxiety when coadministered with AM-251. Thus, the antagonist potentiated the effects of the agonist. The experiment was repeated with a slightly larger WIN-55,212 dose (0.5 mg/kg). Again the agonist had no effects on its own but increased anxiety when coadministered with AM-251 (data not shown).

### The effects of chlordiazepoxide in mice and rats

Chlordiazepoxide dose-dependently reduced anxiety in both mice and rats (Table 1). Anxiolytic doses had no significant effect on locomotion but higher doses suppressed locomotion in both species.

**Table 1:** The effect of the reference compound chlordiazepoxide on the plus-maze behaviour of rats and mice

Group and treatment	Dose (mg/kg)	Closed-arm entries	Time in open arms (%)	Open-arm entries (%)
Rats		12.7 ± 0.9	16.5 ± 2.2	37.9 ± 2.8
Vehicle		12.7 ± 0.9	16.5 ± 2.2	37.9 ± 2.8
Chlordiazepoxide	2	15.6 ± 1.4	30.2 ± 4.3*	49.8 ± 4.7
Chlordiazepoxide	3	14.8 ± 1.3	32.1 ± 3.8*	47.8 ± 2.8*
Chlordiazepoxide	5	6.5 ± 0.4*	13.2 ± 3.9	32.8 ± 5.0
Chlordiazepoxide	10	2.0 ± 0.7*	2.8 ± 2.3*	8.9 ± 5.7*
*F*_4,52_		14.80	9.57	10.59
*P*		< 0.0001	< 0.0001	< 0.0001
Mice		19.2 ± 0.8	13.2 ± 1.7	35.4 ± 3.0
Vehicle		19.2 ± 0.8	13.2 ± 1.7	35.4 ± 3.0
Chlordiazepoxide	5	23.7 ± 2.3	20.5 ± 3.5	38.3 ± 2.3
Chlordiazepoxide	10	21.1 ± 2.3	22.1 ± 3.5	41.9 ± 4.2
Chlordiazepoxide	15	13.8 ± 1.5	31.9 ± 5.3*	59.7 ± 4.0*
Chlordiazepoxide	20	17.4 ± 2.8	31.1 ± 4.3*	54.8 ± 3.2*
Chlordiazepoxide	25	6.1 ± 2.3*	49.4 ± 12.6*	45.6 ± 7.0
*F*_5,51_		8.92	3.96	4.87
*P*		< 0.0001	< 0.005	< 0.001

Chlordiazepoxide dose-dependently reduced anxiety in both mice and rats without affecting locomotion at anxiolytic doses

* *P* < 0.05 at least.

### WIN-55,212 sensitivity of evoked postsynaptic currents

**Fig. 6:**
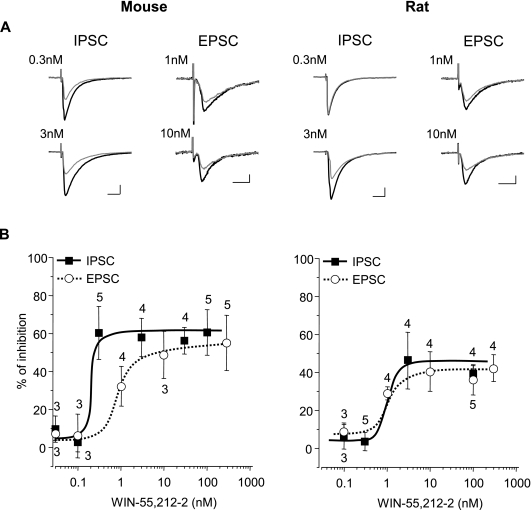
The sensitivity of synaptic inhibition to WIN-55,212 was different in mice and rats. (A) Representative averaged records of 6–9 consecutive events taken before (black) and after 10 min of drug application (grey) are superimposed. The concentration of WIN-55,212 is indicated for each example. (B) Concentration–response relationship of WIN-55,212 on the inhibition of evoked IPSCs and EPSCs recorded in CA1 pyramidal cells in mice and rats. The number of data points at each concentration is indicated above or below the curves for IPSC and EPSCs, respectively. Scale bars, 100 pA and 10 ms for IPSCs, and 50 pA and 10 ms for EPSCs recorded in rats; 25 pA and 10 ms for EPSCs obtained in mice.

Under our conditions, WIN-55,212 was able to reduce the amplitude of both IPSCs and EPSCs by at most half (Fig. 6). In mice, IPSCs were almost one order of magnitude more sensitive than EPSCs to the effects of WIN-55,212. Moreover, IPSCs in mice showed considerably larger sensitivity to WIN-55,212 than did those in rats. In mice, the estimated EC_50_ values of the fitted curves were 0.15 and 0.97 nm for IPSCs and EPSCs, respectively. In rats, estimated EC_50_ values were 0.89 and 0.88 nm for IPSCs and EPSCs, respectively.

In contrast to the difference in pharmacological sensitivity of inhibitory synapses, the properties of IPSCs in control conditions were similar in mice (*n* = 15) and rats (*n* = 16; amplitude, 431.4 ± 62.1 and 431.8 ± 44.8 pA in mice and rats, respectively; *F*_1,29_ = 0.01, *P* > 0.9; rise time, 1.15 ± 0.21 and 1.16 ± 0.35 ms in mice and rats, respectively; *F*_1,29_ = 0.01, *P* > 0.9; decay time constant, 11.8 ± 0.8 and 10.9 ± 0.9 ms in mice and rats, respectively; *F*_1,29_ = 0.41, *P* > 0.5). EPSCs were also similar in the two species (amplitude, 198.1 ± 32.2 and 295.6 ± 38.7 pA in mice and rats, respectively; *F*_1,29_ = 3.76, *P* > 0.05; rise time, 3.5 ± 0.39 and 2.86 ± 0.3 ms in mice and rats, respectively; *F*_1,29_ = 1.73; *P* > 0.1, decay time constant, 12.6 ± 1.3 and 13.9 ± 2.1 ms in mice and rats, respectively; *F*_1,29_ = 0.3, *P* > 0.5).

### The efficacy of WIN-55,212 in hippocampal slices: a note on earlier and present findings

In the present experiments, the effects of WIN-55,212 were similar to those seen earlier but were produced at 100-fold lower concentrations (Hoffman & Lupica, 2000; Hájos & Freund, 2002). Importantly, we used here and in our previous study (Hájos & Freund, 2002) animals from the same source, the same electrophysiological equipment and the same solvent for WIN-55,212. However, the preparation and storage of slices, as well as the flow rate of the solution during recordings, were altered: slices were kept in an interface-type chamber before recordings, and a higher flow rate was used in the recording chamber. This ensured better oxygenation of the tissue (Hájos *et al*., 2005). In addition, the amplitude of evoked synaptic currents became more stable under these conditions. We performed additional experiments to compare endocannabinoid signalling in slices stored in submerged vs. interface chambers. We found not only that the efficacy of WIN-55,212 was markedly increased in the latter, but also that depolarization-induced suppression of inhibition, known to involve CB1 receptors, was more stable and enhanced (N. Hajos, unpublished observations). We believe that the changes in the *in vitro* experimental conditions might better approximate the *in vivo* conditions. It is noteworthy that the present data are in line with those measured in cell cultures (Ohno-Shosaku *et al*., 2002) and binding assays (Felder *et al*., 1995).

### Immunocytochemistry

The general features of CB1 staining in mice and rats have been described in detail earlier (Freund *et al*., 2003; Mátyás *et al*., 2004). Therefore, we focused only on species differences in this study. We stress again that the antibody used here visualized CB1 receptors located on GABAergic neurons (see above).

**Fig. 7:**
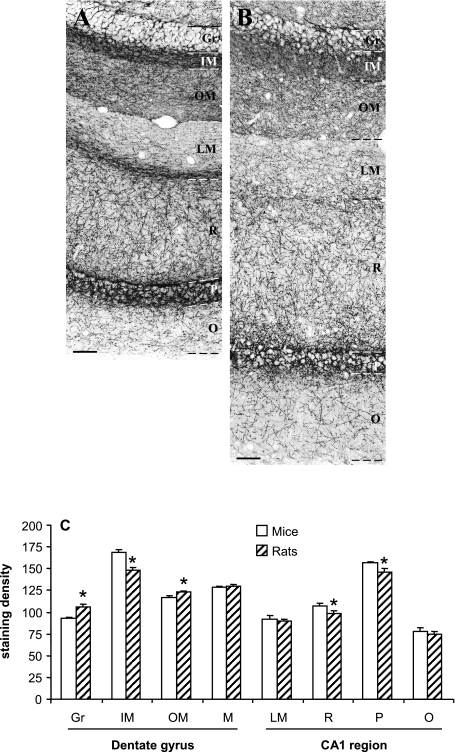
Comparison of CB1 immunorectivity on GABAergic axons and axon terminals in mouse and rat hippocampi. (A and B) Representative photomicrographs showing CB1 staining intensity in mice and rats, respectively; (C) Mouse–rat differences in CB1 staining. Gr, stratum granulosum; M, stratum moleculare as a whole; LM, stratum lacunosum–moleculare; O, stratum oriens. Note that the areas of the inner and outer parts of stratum moleculare are not the same; therefore, staining intensity in the whole area is not equal to the average of inner and outer parts. *P < 0.05, at least, between mice and rats. For statistical details see text. Scale bars, 50 µm (A and B).

In the stratum granulosum of the dentate gyrus, stronger staining was obtained in rats than in mice (*F*_1,8_ = 22.41, *P* = 0.0014; Fig. 7). When the stratum moleculare was taken as a whole, no significant differences occurred (*F*_1,8_ = 0.24, *P* > 0.5). However, the inner third and outer two-thirds of the stratum moleculare showed species-related differences when analysed separately. Staining was stronger in the mouse inner stratum moleculare (*F*_1,8_ = 22.99; *P* = 0.0013). A smaller, but still significant, difference was seen in the outer two-thirds of stratum moleculare, now in favour of rats (*F*_1,8_ = 7.63, *P* = 0.024). In the CA1 region, staining was significantly stronger in the stratum pyramidale of mice than of rats (*F*_1,8_ = 6.07, *P* = 0.039). In the stratum radiatum, the difference was marginally significant (*F*_1,8_ = 3.94, *P* = 0.08).

## Discussion

We found that cannabinoid ligands have opposite effects on anxiety in mice and rats. In mice, the cannabinoid agonist WIN-55,212 produced anxiolytic effects which were readily abolished by the CB1 antagonist AM-251. In rats, the agonist was anxiogenic, an effect that was, surprisingly, potentiated by the antagonist. It is noteworthy that the reference compound used in this study (chlordiazepoxide) dose-dependently decreased anxiety in both species. The relative cannabinoid sensitivity of GABAergic IPSCs and glutamatergic EPSCs also showed marked species differences.

### Comparison with earlier findings

Our study confirms earlier findings on the contrasting effects of cannabinoids in mice and rats. CB1 knockouts as well as wild-type mice treated with AM-251 showed anxiety in various tests, whereas cannabinoid agonists decreased anxiety (Berrendero & Maldonado, 2002; Haller *et al*., 2002, 2004a, 2004b; Maccarrone *et al*., 2002; Martin *et al*., 2002; Valjent *et al*., 2002; Uriguen *et al*., 2004; Rodgers *et al*., 2005; Patel & Hillard, 2006). In addition, the antagonist abolished the effects of the agonist in two studies (Berrendero & Maldonado, 2002; Haller *et al*., 2004a). Thus, our findings in mice are consistent with earlier reports. Conflicting effects were reported with the cannabinoid antagonist SR-141716A (anxiolysis: Haller *et al*., 2002; Rodgers *et al*., 2003; Griebel *et al*., 2005; anxiogenesis: Patel & Hillard, 2006). However, SR-141716A, in contrast to AM-251, affected various functions in CB1-KO mice (including anxiety), suggesting that this antagonist also affects the putative novel receptor (Jarai *et al*., 1999; Breivogel *et al*., 2001; Hájos *et al*., 2001; Haller *et al*., 2002; Kõfalvi *et al*., 2003).

More intriguing findings were, however, obtained with rats. There are virtually no studies investigating the interaction between cannabinoid antagonists and agonists in this species. We show here that AM-251 does not inhibit the anxiogenic effects of WIN-55,212; moreover, it potentiates the effects of low doses that are ineffective *per se*. It is noteworthy that the effect of the agonist was consistent with earlier reports in rats (Arevalo *et al*., 2001; Rodriguez de Fonseca *et al*., 1996; Giuliani *et al*., 2000; Marin *et al*., 2003; Genn *et al*., 2004; Hill & Gorzalka, 2004; Marco *et al*., 2004; McLaughlin *et al*., 2005). Taken together, our findings suggest that WIN-55,212 acts via different mechanisms in the two species.

### Mechanisms of cannabinoid action in mice and rats: a hypothesis

**Fig. 8:**
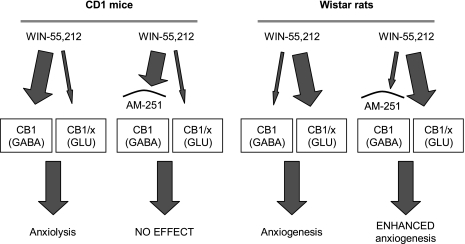
A hypothesis on the mechanisms underlying the differential anxiety-related effects of cannabinoids in CD1 mice and Wistar rats. The agonist is probably acting via CB1 receptors located on GABAergic neurons in CD1 mice. In Wistar rats, the agonist is probably acting via cannabinoid receptors located on glutamatergic (GLU) neurons. As earlier studies suggest that glutamatergic mechanisms are affected by a novel cannabinoid receptor (CBx), but recent studies suggest that the CB1 receptor is also located on glutamatergic cells, either the CB1 or the CBx receptor may mediate the effects of WIN-55,212 on glutamatergic transmission. Differences seen here between CD1 mice and Wistar rats may also exist between different mouse and possibly rat strains (see Discussion).

Cannabinoids retrogradely inhibit both glutamate and GABA release (Hájos *et al*., 2001; Marsicano & Lutz, 1999; Hájos & Freund, 2002; Piomelli, 2003; Katona *et al*., 2006), which play opposite roles in anxiety (Millan, 2003). We hypothesize that discrepant behavioural findings are explained by a differential cannabinoid sensitivity of glutamatergic and GABAergic neurotransmission in Wistar rats and CD1 mice (Fig. 8).

As shown earlier, WIN-55,212 inhibits both IPSCs and EPSCs in the hippocampus (Hájos & Freund, 2002). Thus, the agonist affects both GABA and glutamate neurotransmission. AM-251 abolished the WIN-55,212-induced inhibition of IPSCs but not of EPSCs, strongly suggesting that this antagonist is active on GABAergic but not on glutamatergic neurons. The discrepancy might be related to the distinctive features of CB1 receptors located on GABAergic and glutamatergic neurons (Katona *et al*., 2006). Taken together, these considerations suggest that AM-251 inhibits those effects of WIN-55,212 mediated by GABAergic but not glutamatergic neurons.

In mice, anxiety appears to be decreased by WIN-55,212 via CB1 receptors located on GABAergic neurons, as the effect was readily abolished by AM-251. We suggest that, in rats, the WIN-55,212-induced anxiety was mediated by glutamatergic mechanisms, as AM-251 did not inhibit this effect. Nevertheless, one can assume that WIN-55,212 still affected GABA neurotransmission in rats, which may explain the antagonist-induced potentiation of the agonist effect (Fig. 8). When the antagonist and agonist were administered together in rats, the GABA-mediated anxiolysis was probably inhibited by AM-251, which enhanced glutamate-mediated anxiogenesis. Our hypothesis involves glutamate-mediated anxiogenesis by cannabinoids being stronger than GABA-mediated anxiolysis. Earlier findings with SR-141716A indirectly support this assumption. The effects of SR-141716A on glutamatergic functions persisted in CB1-KO mice, but its effects on GABAergic mechanisms disappeared in this genotype (Hájos *et al*., 2001; Kõfalvi *et al*., 2003). However, SR-141716A decreased anxiety in both CB1-KOs and wild types, suggesting that its glutamatergic effects were more important for anxiety than its GABAergic effects (Haller *et al*., 2002). It is noteworthy that SR-141716A, unlike AM-251, inhibited the effects of WIN-55,212 on both IPSCs and EPSCs.

Our hypothesis is supported by studies concerning the effects of cannabinoids on baroreceptor input into the n. tractus solitarius (Rademacher *et al.*, 2003; Seagard *et al*., 2004). In dogs, the effects of cannabinoids appeared to be mediated by glutamatergic mechanisms, whereas both GABAergic and glutamatergic mechanisms were operational in rats. Patel & Hillard (2006) also suggested that different cannabinoid agonists may induce different signalling pathways although they activate the same receptor.

The electrophysiological study strengthened the view that the CB1 receptors involved in the anxiolytic effect of WIN-55,212 were located on GABAergic neurons in mice, as IPSCs were about one order of magnitude more sensitive to the action of this compound than were EPSCs. Naturally, differences in the sensitivity to WIN-55,212 became meaningless if receptors were fully occupied, i.e. both IPSCs and EPSCs were maximally inhibited (note that the magnitude of the effect was similar for the two currents). However, it has been shown that the behavioural effects of cannabinoids are produced at very low receptor occupancy (Gifford *et al*., 1999). In addition, dose–response studies for various behavioural effects of WIN-55,212 show that the maximal effect is expressed at doses higher than 3 mg/kg (rats: De Vry *et al*., 2004; mice: Darmani, 2001; Janoyan *et al*., 2002). Moreover, the lowest dose that induced significant effects was as high as 20 mg/kg in some tests. Taken together, these data preclude cannabinoid receptors being fully occupied during behavioural experiments and render the IPSC–EPSC difference in WIN-55,212 sensitivity meaningful.

In rats, WIN-55,212 affected IPSCs and EPSCs with a similar efficacy. As the compound was anxiogenic in rats but the two currents were equally affected, one has to conclude that cannabinoid effects mediated by glutamatergic neurotransmission can override those mediated by GABAergic mechanisms in anxiety tests (see above). Taken together, our findings suggest that the anxiety-related effects of cannabinoids depend on the relative cannabinoid sensitivity of GABAergic and glutamatergic neurotransmission. When the former prevails (e.g. in CD1 mice), cannabinoid agonists decrease anxiety. When glutamatergic and GABAergic neurotransmission are equally affected (e.g. in Wistar rats), the same agonist increases anxiety.

Conclusions based on behavioural findings were tested here by electrophysiological studies performed in the hippocampus, a brain region that is strongly involved in the control of anxiety (McNaughton, 1997; File *et al*., 2000). Nevertheless, fear and anxiety processing is not restricted to this brain region. In addition, electrophysiological experiments were performed in neonatal rodents (for technical reasons) whereas behavioural studies were performed in young adults. Nevertheless, this is the first study showing that GABAergic and glutamatergic sensitivity to cannabinoids is different in mice and rats. One can hypothesize that similar differences exist in adult rats, have an impact on the actions of cannabinoids and underlie their differential effect on anxiety.

In certain CA1 subregions, CB1 receptor expression was lower in rats than mice. However, differences were small. In addition, the expression of CB1 receptors was larger in rats when certain subregions of the dentate gyrus were considered. Taken together, these findings suggest that receptor expression levels had a minor impact on species differences in relative cannabinoid sensitivity. Such differences might be explained by other phenomena, e.g. by differential coupling efficacies at GABAergic terminals or by differences in tonic brain endocannabinoid levels. The latter assumption is supported by the differential effect of AM251 in mice and rats. One can hypothesize that the endocannabinoid tone is involved in setting the level of transmission through GABAergic and glutamatergic synapses in the hippocampus. The putative novel cannabinoid receptor may also be involved. It has been repeatedly shown that CB1-KO mice respond to cannabinoid treatments in a variety of experimental conditions (Breivogel *et al*., 2001; Jarai *et al*., 1999; Zimmer *et al*., 1999; Hájos *et al*., 2001; Haller *et al*., 2002; Kõfalvi *et al*., 2003). These residual effects of cannabinoids are unlikely to be due to the background strain used for generating the knockout strain, or to errors in it, as they were shown in two different CB1-KO lines generated by Ledent *et al*. (1999) and Zimmer *et al*. (1999). Earlier, we assumed that glutamatergic mechanisms are affected by cannabinoids via a novel cannabinoid receptor. The main support for this conclusion was that the GABAergic effects of cannabinoids disappeared in CB1-KO mice, whereas glutamatergic effects persisted (Hájos *et al*., 2001; Kõfalvi *et al*., 2003). However, the expression of the novel cannabinoid-sensitive site may have been induced by the knockout of CB1, as a compensatory mechanism, while in wild types CB1 could still be the predominant receptor controlling glutamate release (Marsicano & Lutz, 1999; Katona *et al*., 2006). Therefore, the identity of receptors targeted by WIN-55,212 at glutamatergic synapses remains to be established. At the present stage, however, one cannot exclude the possibility that a novel receptor was involved in the species differences shown above.

### Consequences for cannabinoid physiology

The findings of the present study suggest that the behavioural effects of cannabinoids depend on the relative responsiveness to cannabinoids of different, often antagonistic, transmitter systems or neuron types. In this regard, a major difference was found between CD1 mice and Wistar rats. Nevertheless, disparate studies suggest that similar differences may exist between different mouse strains as well. For example, Akinshola *et al*. (1999) showed large strain differences in the anxiety-related effects of SR-141716A (the strains studied were ICR, C57/BL6 and DBA2). In our studies, the same antagonist was anxiolytic in maze-naive CD1 mice (Haller *et al*., 2002) whereas in the study by Rodgers *et al*. (2003) similar effects were obtained only in maze-experienced but not maze-naive Swiss–Webster mice. In addition, AM-251 blocked the effect of WIN-55,212 on glutamate release in Sprague–Dawley but not in Wistar rats (Hájos & Freund, 2002; Hoffman *et al*., 2005). These discrepancies suggest that differences seen here between CD1 mice and Wistar rats may also exist between different mouse and possibly rat strains.

### Conclusions

The cannabinoid agonist WIN-55,212 was anxiolytic in CD1 mice and anxiogenic in Wistar rats. These effects are largely consistent with earlier findings. The effects of the agonist were readily abolished by AM-251 in mice. Surprisingly, however, AM-251 potentiated the effects of WIN-55,212 in rats. The species-dependent effects of the antagonist, as well as our electrophysiological studies, suggest that WIN-55,212 affects anxiety primarily via GABAergic mechanisms in CD1 mice and predominantly via glutamatergic mechanisms in Wistar rats.

## Abbreviations

ACSF: artificial cerebrospinal fluid
CB1-KO: cannabinoid CB1 knockout
EPSC: excitatory PSC
GABA: gamma-aminobutyric acid
IPSC: inhibitory PSC
PSC: postsynaptic current
